# Identification of Potential Key Agents for Targeting RNA-Dependent RNA Polymerase of SARS-CoV-2 by Integrated Analysis and Virtual Drug Screening

**DOI:** 10.3389/fgene.2020.581668

**Published:** 2020-11-17

**Authors:** Shuang Ao, Dan Han, Lei Sun, Yanhong Wu, Shuang Liu, Yaojiang Huang

**Affiliations:** ^1^Beijing Engineering Research Center of Food Environment and Public Health, Minzu University of China, Beijing, China; ^2^College of Medicine, Minzu University of China, Beijing, China; ^3^Beijing Wildlife Conservation and Natural Reserve Management Station, Beijing Gardening and Greening Bureau, Beijing, China; ^4^College of Life and Environmental Sciences, Minzu University of China, Beijing, China; ^5^Harvard T.H. Chan School of Public Health, Boston, MA, United States

**Keywords:** SARS-CoV-2, COVID-19, family analysis, molecular docking, virtual drug screening

## Abstract

**Background:**

RNA-dependent RNA polymerase (RdRp) is the key enzyme responsible for the SARS-CoV-2 replication process and catalyzes the synthesis of complementary minus strand RNA and genomic plus strand RNA, often recognized as good targets for antiviral drugs.

**Materials and Methods:**

A systematic screening of existing antiviral compounds, family analysis, conserved domain analysis, three-dimensional structure modeling, drug virtual screening, and bioassays were performed to identify agents that potentially targeted RNA-dependent RNA polymerase of SARS-CoV-2.

**Results:**

Four thousand nine hundred and forty seven antiviral lead compounds were selected and evaluated by systematic screening. Of these, 359 agents were screened by family analysis and conserved domain analysis. They were further analyzed by three-dimensional structure modeling, virtual drug screening, and bioassays. The results identified 102 agents with potential for repurposing to target the RNA-dependent RNA polymerase of SARS-CoV-2.

**Conclusion:**

This study identified 102 key agents with potential anti-SARS-CoV-2 RNA-dependent RNA polymerase function and prospects of rapid clinical application for the treatment of COVID-19.

## Introduction

The COVID-19 has spread to 188 countries and regions worldwide with 30 million infections and 950,000 deaths (World Health Organization [WHO], 2020a). COVID-19 is caused by the novel severe acute respiratory syndrome coronavirus 2 (SARS-CoV-2), which is a positive-sense, single-stranded beta-coronavirus. The SARS-CoV-2 RNA genome (∼29,903 nucleotides) (MN908947.3) encodes non-structural, structural, and accessory proteins (NC_045512.2) (Wu et al., 2020b). RNA-dependent RNA polymerase (RdRp) (YP_009725307.1) is a non-structural protein and one of a key enzyme responsible for viral replication. It is responsible for synthesis of a complementary minus strand RNA and genomic plus strand RNA (Wu et al., 2020a; Zhu et al., 2020), and is recognized as a good target for antiviral drugs (Zumla et al., 2016; Gordon et al., 2020a).

So far there are no small-molecule drugs, vaccines, or monoclonal antibodies approved for the treatment of COVID-19. The priority for the emerging COVID-19 epidemic is to prevent wider spread and develop a vaccine or a drug (Zhu et al., 2020). However, the development of new drugs or new vaccines for new infectious diseases requires many months or years (Bedford et al., 2019). Given the severity of COVID-19 outbreak around the world, to rapidly identify and repurpose existing agents or compounds against SARS-CoV-2 in short amount of time is another option (Farha and Brown, 2019; Li and De Clercq, 2020).

The DrugBank^1^, Therapeutic Target Database (TTD)^2^, ChEMBL^3^, and Binding Database^4^ database were searched by RdRp name and sequence to screen potential agents or compounds that were known agents or compounds targeting other coronaviruses (Lu et al., 2006; Tsai et al., 2006; Heidebrecht et al., 2009; Lee et al., 2009; Kumar et al., 2016) and may act on proteins or genes of SARS-CoV-2. Then, we performed integrated analysis and drug virtual screening to identify potential key lead compounds that targeted the RdRp of SARS-CoV-2.

## Materials and Methods

### Data Screening, Download, and Processing

The raw data were systematically searched using RdRp name and sequence through TTD (see text footnote 2), DrugBank (see text footnote 1), ChEMBL (see text footnote 3), and The Binding Database (see text footnote 4). The relevant data files were downloaded and saved in the CSV and SDF formats. The datasets were merged, records of duplication or missing key information were removed, and the CHEMBL IDs were converted to ZINC ID or PubChem IDs using RStudio version 1.2.5019 (RStudio, Inc., 2009–2018) (RStudio Team, 2015) and PubChem Identifier Exchange Service.

### Biosequence Analysis and Conserved Domain Analysis

Protein family analysis was performed by the Biosequence analysis on the HMMER web server^5^ and Conserved Domain analysis using the NCBI Conserved Domains web server^6^, to evaluate search results that had conserved domains similar to SARS-CoV-2 RdRp. The similarity of protein sequences search use the default value in the database, the threshold of sequence similarity in the conservative region selects the default value (80%).

### Structure Modeling

The three-dimensional structure of RdRp were simulated and generated by using Discovery Studio 2016 [v 2016] (Biovia Dicovery Studio, 2015) and SWISS-MODEL online server (Biasini et al., 2014). The four steps of the process included identification of template(s), alignment, model-building, and evaluation. With a score between 0 and 1, a higher GMQE score indicates the higher the reliability. The QMEAN score (between −4.0 and 0) indicated accuracy and reliability of the model. If SARS-COV-2 RdRp was constructed in 3D by electron microscopy or X-ray crystallography, the 3D structure was downloaded from the PDB database^7^.

### Virtual Drug Screening

The three-dimensional structure file of SARS-CoV-2 RdRp was downloaded in SDF format from PubChem or ZINC website. We performed molecular docking and virtual drug screening using Autodock Vine (Trott and Olson, 2010) and PyRx (Dallakyan and Olson, 2015) for rapidly identifying lead compounds to target SARS-CoV-2 RdRp. the steps included loading proteins and ligands, making macromolecule and ligand, Running AutoGrid, Molecular docking, Analyzing, Exporting Results, Virtual Screening, and BioAssay. The structure modeling and virtual drug screening results were viewed and analyzed using Pymol.

### BioAssay, Molecular Property, and Cluster

The candidate drugs were screened had been subjected to activity experiments, and the target protein of these activity experiments was similar to the SARS-CoV-2 RdRp protein. The candidate drugs were analyzed and screened by analyzing the results of bioassays (such as IC50 determination, inhibition assay, and Ki expression), computing basic molecular property, structure format interconversions, and clustering identical or very similar compounds using ChemmineR and ChemmineOB in R (RStudio, Inc., 2009–2018) (RStudio Team, 2015).

## Results

### Data Processing and Screening

A total of 4947 compounds were found by systematic retrieval, of which 32 were recorded from TTD and DrugBank, 2964 were recorded from the ChEMBL database, and 1955 were retrieved from the Binding Database. The three data frames are named as BT, ChE, and BD (Supplementary Table S1–S3).

### Potential Repurposing Agent Screening

The three data frames of BT, ChE, and BD were merged and the retrieved records with duplication and missing key information were removed using R software. By family and conservative domain analysis, compounds acting on a protein who similar to SARS-CoV-2 RdRp in conserved region and structure were retained, instead, proteins and corresponding compounds in structure or conserved domain that were not similar to SARS-CoV-2 protein wrere excluded. Family analysis was performed using the HMMER web server and conserved domain analysis was done using the NCBI web server to determine whether the target sequence and structure of the selected agents were similar to SARS-CoV-2 RdRp sequence and structure. SARS-CoV-2 RdRp sequence matched to the family Corona_RPol_N (pfam06478.13) and RdRP_1 (pfam00680.20). RNA-dependent RNA polymerase of Hepatitis C Virus (O39930) and RNA-directed RNA polymerase L of HPIV-2 (P26676), among others, were excluded as they did not belong to the same family (pfam06478.13, pfam00680.20) and lacked conserved domains (Corona_RPol_N) similar to SARS-CoV-2 RdRp. A total of 359 unique agents that would potentially target severe acute respiratory syndrome coronavirus (SARS-CoV), Middle East respiratory syndrome coronavirus (MERS-CoV), and feline coronavirus (FCoV) were screened for potential repurposing SARS-CoV-2 RdRp (Supplementary Table S4).

### Structure Modeling

**FIGURE 1:**
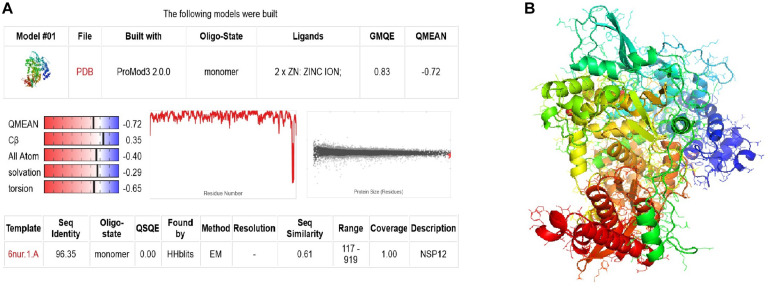
The structure modeling of SARS-CoV-2 RdRp. **(A)** The homology modeling report of RdRp. **(B)** The modeling 3D structure of RdRp.

The three-dimensional structure model for SARS-CoV-2 RdRp was formed using Discovery Studio 2016 [v. 2016] and SWISS-MODEL online server (Biasini et al., 2014). NSP12 (6nur.1.a) SARS-CoV was selected as the template for protein modeling. The result showed that the sequence identity was 96.35% and the QMEAN score was −0.72, which indicated a good agreement between the model structure and experimental structures of similar size (Figure 1A). The 3D structure of the model is shown in Figure 1B.

### Drug Virtual Screening

**FIGURE 2:**
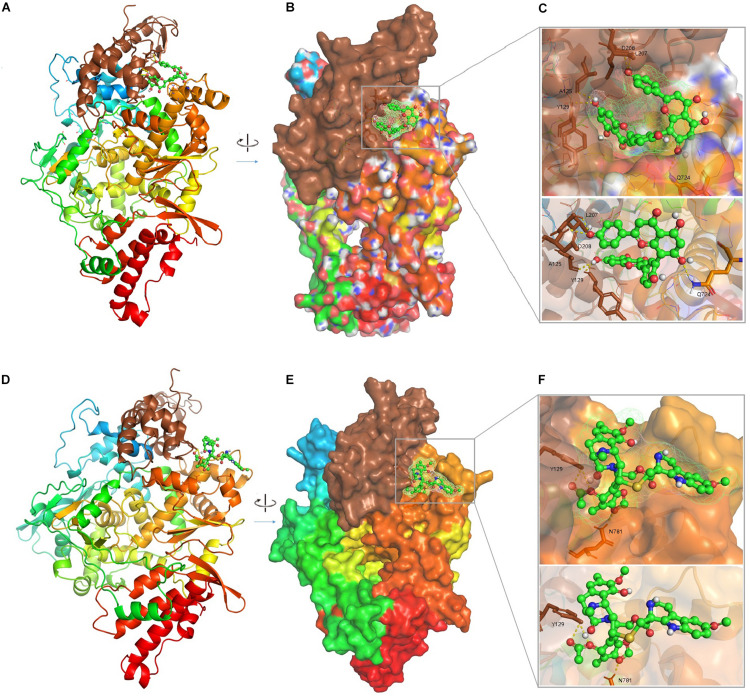
The complex 3D structure of SARS-CoV-2 RdRp with potential agents. **(A)** Cartoon representation of the RdRp-inhibitor (Amentoflavone, PubChemCID 5281600) complex. **(B)** Surface representation of the RdRp-Amentoflavone complex. **(C)** An enlarged view of the RdRp substrate-binding pocket with Amentoflavon. The key residues 125A, 129Y, 208D, 207L, and 724Q are shown as brown sticks; the background is the surface of RdRp substrate-binding pocket; Amentoflavon is shown as green and red spheres and sticks. **(D)** Cartoon representation of the RdRp-inhibitor (Lurbinectedin, PubChemCID 57327016) complex. **(E)** Surface representation of the RdRp-Lurbinectedin complex. **(F)** An enlarged view of RdRp substrate-binding pocket with Lurbinectedin. The key residues 781N and 129Y are shown as brown sticks; the background is the surface of substrate-binding pocket of RdRp; Lurbinectedin is shown as green and red spheres and sticks.

**FIGURE 3:**
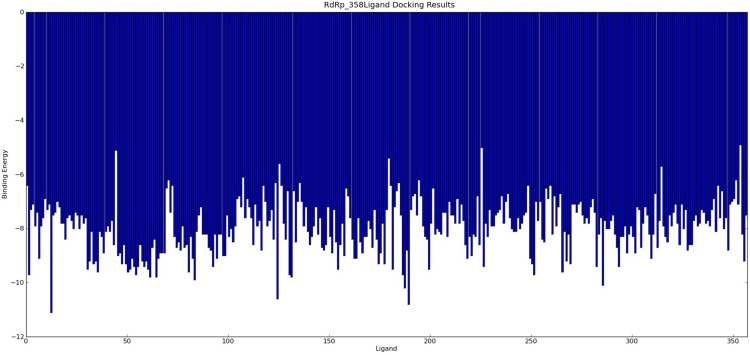
358 agents Docking Results with RdRp. The binding energy of ligand and RdRp were −4.9 to −11.1.

The agents targeting SARS-CoV, MERS-CoV, and FCoV (Supplementary Table S4) were used for molecular docking with RdRp and potential candidates for repurposing for SARS-CoV-2 RdRp were identified by virtual drug screening. The results showed 358 potential agents that fitted tightly into the RdRp binding pocket (Figure 2). However, ligand drug No. 13 (ISIS 2922/Formivirsen sodium) was excluded due to its high molecular weight and unsuitability as a ligand. AutoDock is suitable for small molecular drugs and proteins, and is not suitable for docking between macromolecules and proteins. The binding energy or binding affinity of RdRp and ligand were −4.9 to −11.1. A lower binding energy indicates a more stable ligand receptor. The 300 lead agents had binding energies lower than −7 to RdRp (Figure 3 and Supplementary Table S4). These agents could be used as potential repurposing candidates to target the RdRp of SARS-CoV-2.

### Bioassay, Molecular Property, and Cluster

Bioassay results showed that most candidate drugs had the values of IC50 and inhibition but lacked Ki values (Supplementary Table S4). For the drugs derived from DrugBank or TDD database, although there was no IC50 or inhibition data, they were still included in the selected drugs because these drugs had already been approved or investigated for other viruses. We screened candidates with IC50 less than 10,000 or inhibition % more than 50, or drugs that were approved or investigated for treatment of other viral infections, and a total of 102 drugs were selected (Supplementary Table S5).

**FIGURE 4:**
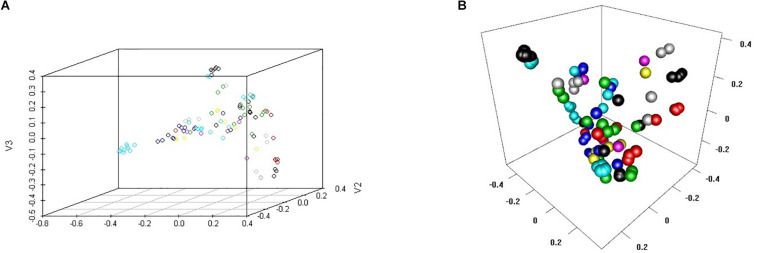
MDS cluster scatter plot and Interactive 3D scatter plot. **(A)** 3D scatter plot of Multi-Dimensional Scaling (MDS). **(B)** Interactive 3D scatter plot to visualize and compare clustering results.

**FIGURE 5:**
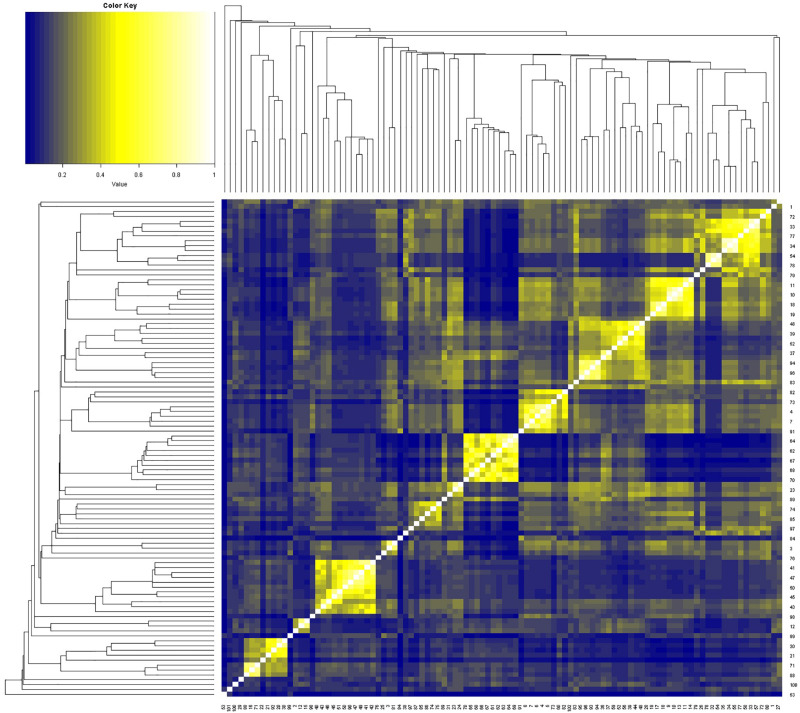
The 102 drugs were visualized and compared as clustering results in plot dendrogram with heatmap. A lower score and a darker color indicated an increasing distance.

These 102 drugs underwent basic molecular property analysis, multi-dimensional scaling (MDS) and interactive 3D scatter plot (Figure 4). The results were visualized in a plot dendrogram with a heatmap to analyze and compare clustering results with identical or very similar compounds obtained by ChemmineR and ChemmineOB (Figure 5). Plot heatmap with dendrogram and Hierarchical clustering and atom pair distance matrix. The color represents the clustering score and distance, the color gets darker and the score gets lower when the distance gets farther, instead, the color is white and the score is 1, which means it is same compound. Heatmap results showed that the 102 agents were relatively independent in hierarchical clustering by atom pair distance, and these agents could be used as candidate agents for clinical trials. As the color got darker and the scores got lower as the distance between the agent and enzyme increased. The white color and the score = 1 indicated the same compound.

Of the 102 agents screened, five were approved for treatment of different diseases (Sofosbuvir, Rifamycin, Baloxavir Marboxil, Rifampin and Rifapentine), two were undergoing clinical trials (Remdesivir and Lurbinectedin) and most other agents have been experimentally studied. Activity studies have shown that Sofosbuvir acts as modulator, Rifapentine acts as binder, and most of the others are shown to be virus inhibitors. Their target disease or target organism, respectively, Zaire ebolavirus (Remdesivir), Hepatitis C virus infection (Sofosbuvir and Rifamycin), Influenza virus infection (Baloxavir marboxil), Tuberculosis (Rifampin and Rifapentine) and FCoV (CHEMBL4202812 and CHEMBL4204431), others mostly used for MERS-CoV or SARS-CoV.

## Discussion

The COVID-19 has caused a global public health emergency and become a worldwide pandemic (World Health Organization [WHO], 2020b). The top priority is to develop vaccines and drugs that can effectively prevent or cure viral infections. Nevertheless, the development of new antiviral drugs or new vaccines for clinical use require many months, and sometimes years (Bedford et al., 2019). A quick and good alternative can be the application or repurposing of existing antiviral drugs or compounds to rapidly identify potential drugs candidates against SARS-CoV-2 infections (Farha and Brown, 2019; Cohen, 2020).

SARS-CoV-2 RdRp are non-structural proteins and have high structural homology with MERS-CoV and SARS-CoV. RdRp plays a key role in the viral life cycle and is responsible for the synthesis of complementary minus strand RNA and genomic plus strand RNA. In the absence of any of these RNA components, the virus is unable to proliferate in host cells or cause disease, therefore, RdRp is considered a good target for antiviral drugs (Zumla et al., 2016; Gordon et al., 2020a).

We performed a systematic search by sequence and name, integrated analysis, structure modeling, and virtual drug screening to identify existing antiviral drugs or compounds that had the potential to become key lead compounds targeting RdRp of SARS-CoV-2. First, a total of 4947 compounds were selected from the TTD, DrugBank, Binding Database, and ChEMBL Database by systematic search. Then, 359 unique agents were selected for potential repurposing by family analysis and conserved domain analysis. We selected and recommended drugs based on relatively small IC50, or inhibition rate of more than 50%, or drugs that were under investigation or approved. Finally, 102 agents for SARS-CoV-2 RdRp as a target were identified by structure modeling, drug virtual screening, and they have been analyzed by bioassays (Supplementary Table S5). The results showed that the approved drugs, including sofosbuvir, rifamycin, baloxavir marboxil, rifampin, rifapentine, and drugs under investigation like Remdesivir (RDV) and Lurbinectedin, had low binding energies with RdRp and were potential inhibitors of RdRp. In previous studies, RDV, a nucleotide analog inhibitor of RdRp, showed extensive antiviral activity against RNA viruses, including MERS-CoV and SARS–CoV (Gordon et al., 2020a,b). Given the high amino acid sequence and structural similarities between SARS-CoV-2, SARS-CoV, and MERS-CoV RdRps, these antiviral agents would also inhibit the SARS-CoV-2 RdRps. We identified a set of existing antiviral agents with clinical potential for the treatment of SARS-CoV-2 infection. These results may provide guidance for the generation of more potent anti-SARS-CoV-2 agents.

This study has achieved some meaningful results, it could also have some limitations. A set of antiviral drugs with a potential for clinical use against SARS-CoV-2 were founded, but these agents have been used in different experimental or investigational studies or already been approved (Heidebrecht et al., 2009; Lee et al., 2009; Kumar et al., 2016). However, the studies conducted with these agents were for other viruses that were very similar in sequence and structure to SARS-CoV-2. Further studies are need to determine the pharmacodynamics and specificity of the anti-SARS-CoV-2, preclinical studies, clinical trials of drugs, and so on. However, we first screened which compounds had functions on proteins with similar structure to SARS-CoV-2 RdRp (proved in experimentally or clinically), and then conservative domain analysis. On this basis, we performed molecular docking and drug screening. This has an experimental or clinical basis, molecular docking and drug screening is more reliable. Studies indicated that the structure-based molecular docking is useful, which are an important part of the drug discovery duo to it use for rapid drug screening using receptor-ligand interaction energy and structural optimization (Macalino et al., 2015; Jin et al., 2020). Meanwhile, multiple approaches such as machine learning and transcriptomic data approaches are developing (Aliper et al., 2016; Klambauer et al., 2019).

## Conclusion

In conclusion, 4947 antiviral agents were selected by systematic screening of existing antiviral compounds, of which 359 agents were screened by family analysis and conserved domain analysis. Finally, 102 agents that had the potential to be repurposed for anti-SARS-CoV-2 RdRp by drug virtual screening and bioassays. This study identified key virus-targeting agents that may be future lead compounds for rapid clinical use in the treatment of COVID-19 and anti-SARS-CoV-2.

## Data Availability Statement

All datasets presented in this study are included in the article/Supplementary Material.

## Author Contributions

YH proposed the idea and design of the study, had full access to all data in the study, and were responsible for the integrity of all data and the accuracy of the data analysis. SA, YW, LS, and DH contributed to the raw data acquisition and writing of the manuscript. SA, YW, and YH contributed to important revisions of the manuscript. SA, DH, LS, and YH contributed to the statistical analysis. All authors participated in data acquisition, data analysis, or data interpretation, and reviewed and approved the final version.

## Conflict of Interest

The authors declare that the research was conducted in the absence of any commercial or financial relationships that could be construed as a potential conflict of interest.

## Footnotes

1 https://www.drugbank.ca/

2 http://db.idrblab.net/ttd/

3 https://www.ebi.ac.uk/chembl/

4 https://www.bindingdb.org/

5 https://www.ebi.ac.uk/Tools/hmmer/

6 https://www.ncbi.nlm.nih.gov/Structure/cdd/wrpsb.cgi

7 http://www.rcsb.org

## Supplementary Material

Supplementary Table 1 List of Compounds selected from TTD and DrugBank.

Supplementary Table 2 List of Compounds selected from ChEMBL.

Supplementary Table 3 List of Compounds selected from Binding Database.

Supplementary Table 4 List of unique virus-targeting agents with potential repurposing for SARS-CoV-2 RdRp.

Supplementary Table 5 Virus-targeting agents as repurposed candidates for potential clinical use against SARS-CoV-2 RdRp.

## Abbreviations

COVID-19: coronavirus disease 2019
WHO: the World Health Organization
SARS-CoV-2: severe acute respiratory syndrome coronavirus 2
RdRp: RNA-dependent RNA polymerase
TTD: therapeutic target database
SARS-CoV: severe acute respiratory syndrome coronavirus
MERS-CoV: Middle East respiratory syndrome coronavirus
FCoV: feline coronavirus
IC50: half maximal inhibitory concentration
Ki: inhibition constant.
